# Treatment‐Related Adverse Event Claims in Otorhinolaryngology—Head and Neck Surgery: A Nationwide Finnish Review (2012–2023)

**DOI:** 10.1111/coa.70114

**Published:** 2026-04-21

**Authors:** Alexander Westerholm, Satu Lamminmäki, Lasse Rämö, Leena‐Maija Aaltonen, Paula Virkkula, Antti Mäkitie, Morag Tolvi

**Affiliations:** ^1^ Department of Otorhinolaryngology – Head and Neck Surgery Helsinki University Hospital and University of Helsinki Helsinki Finland; ^2^ Department of Orthopedics and Traumatology University of Helsinki and Helsinki University Hospital Helsinki Finland; ^3^ Patient Insurance Centre Helsinki Finland; ^4^ Research Program in Systems Oncology, Faculty of Medicine University of Helsinki Helsinki Finland

**Keywords:** complication, head and neck surgery, injury claim, otorhinolaryngology, patient injury, patient safety

## Abstract

**Background:**

This study investigated the frequency and characteristics of patient injuries related to Otorhinolaryngology—head and neck surgery (ORL‐HNS) in Finland from 2012 to 2023, with a focus on common complications, trends in injury frequency and comparisons with previous studies.

**Methods:**

Data were obtained from the Patient Insurance Centre (PIC) of Finland, encompassing all ORL‐HNS‐related patient injury cases from 2012 to 2023. The study reviewed 1153 claims, of which 317 (27.5%) were compensated. The analysed variables included patient demographics, procedure types, complications and healthcare settings. Descriptive statistics were used to summarise the data.

**Results:**

Most compensated injuries (66.6%) were due to surgical or procedural complications, followed by inadequate management (24.3%). Most cases occurred at university hospitals (40.1%) or regional hospitals (38.8%) and were handled by specialists (69.7%). Endoscopic sinus surgery and septoplasty had compensation ratios of 0.7 and 0.72 per 1000 procedures, respectively. The likelihood of compensation decreased with patient age but was not correlated with hospital type. Among subspecialties, rhinology (31%) and head and neck surgery (30%) had the highest frequency of compensated claims.

**Conclusions:**

This study corroborates previous findings that ORL‐HNS patient injuries are relatively rare, with many occurring during routine surgeries performed by specialists. These results challenge the assumption that surgical errors stem primarily from inexperience. The greater number of reported complications in university hospitals may reflect the trend towards centralising complex procedures.

**Level of Evidence:**

4.

## Key Points

- The majority of compensated ORL‐HNS patient injuries in Finland between 2012 and 2023 arose from surgical complications, particularly during routine procedures such as FESS, septoplasty and tonsillectomy.
- Nearly one‐quarter of compensated cases were due to inadequate diagnostics or treatment, underscoring the importance of careful preoperative work‐up and evidence‐based decision‐making.
- Wrong‐site surgery, though rare, persists and must be considered ‘never events’—reinforcing strict verification at every stage of surgical care.
- Most of the compensated cases involved specialists in university or regional hospitals, reflecting the centralisation of high‐risk surgery and showing that complications arise even in expert hands.
- Despite advances in technology, the spectrum of compensated complications has remained unchanged over time, highlighting enduring risks in ORL‐HNS surgery.

## 1 Introduction

Investigating patient injuries is essential for improving healthcare safety and reducing global costs. The World Health Organisation (WHO) has estimated that up to 1 in 10 patients are harmed during hospital care [1]. Preventable medical errors are estimated to cost $17–29 billion in the U.S. annually [2]. Globally, medication errors alone account for an economic burden of $42 billion annually, which is nearly 1% of total health expenditure [3].

Most ORL‐HNS complications arise from surgery (59%) [4], followed by diagnostic (25%) and treatment delays (19%). Surgical and diagnostic failures remain key issues in head and neck surgery and rhinology.

In Finland, all patient injury claims are handled centrally by the Patient Insurance Centre (PIC), ensuring equal national standards. Approximately 24% of claims are compensated (Annual Report 2023) [5].

Recent advances in equipment and changing patient demographics warrant reassessment of complication patterns. Additionally, more procedures are now performed as day surgeries and under local anaesthesia, particularly in ORL‐HNS [6, 7].

This study investigated ORL‐HNS related patient injuries in Finland from 2012 to 2023 and compared them to previously reported findings. Understanding these trends can guide preventive measures and improve care quality and safety for patients and providers alike.

## 2 Materials and Methods

### 2.1 Design

This was a retrospective, registry‐based observational study of patient injury claims related to Otorhinolaryngology—Head and Neck Surgery (ORL‐HNS) in Finland between January 2012 and December 2023.

### 2.2 Setting

Data were obtained from the nationwide PIC.

### 2.3 Participants

The dataset included all ORL‐HNS‐related patient injury decisions made by the PIC during the study period (*n* = 1153). Of these, 317 (27.5%) were compensated. The data comprised patient records, compensation decisions, and healthcare provider statements.

### 2.4 Main Outcome Measures

The primary outcomes were the types and frequencies of complications leading to compensation. The following variables were analysed: patient age and sex, diagnosis, procedure or treatment performed, complication type, provider background (profession, level of training), hospital type and compensation amount.

### 2.5 Ethical Considerations

The study protocol was approved by the institutional review board of the PIC. As a retrospective registry‐based study with no patient intervention, approval from a medical ethics committee and individual informed consent were not required under Finnish legislation (Medical Research Act 488/1999) [8].

### 2.6 Reporting Guideline

The manuscript was prepared in accordance with the STROBE (Strengthening the Reporting of Observational Studies in Epidemiology) guideline for observational studies.

### 2.7 Statistical Analyses

The data processing and analysis were conducted via Microsoft Excel version 16.83 (Microsoft Corp., Redmond, WA, USA). The chi‐square test was used to examine whether there was an association between hospital type (regional vs. university hospitals) and the likelihood of compensation in complaints. Logistic regression analysis was used to assess whether age was associated with the number of compensated complications. Statistical significance was determined with a *p*‐value threshold of 0.05.

## 3 Results

From 2012 to 2023, a total of 1153 ORL‐HNS‐related patient injury claims were filed, of which 317 (27.5%) resulted in compensation. Patient characteristics are presented in Table 1. Patients in compensated cases were more often female (59.9% vs. 55.1% of non‐compensated cases), with a mean age of 47 years (range 0–91 years). Most incidents occurred in university hospitals (40.1%) and were handled by specialist doctors (69.7%).

**TABLE 1:** Characteristics of patients and healthcare providers in 317 ORL‐HNS‐related compensated injuries in Finland from 2012 to 2023.

	Compensated		Not compensated	
	*N*	%	*N*	%
Patient				
Age (range)				
0–15	29	9.1	45	5.4
16–30	42	13.2	122	14.7
31–45	78	24.6	168	20.2
46–60	77	24.3	219	26.4
61–75	71	22.4	219	26.4
76–91	20	6.3	57	6.9
Sex/Female	190	59.9	461	55.1
Hospital				
University Hospital	127	40.1	373	44.6
Regional Hospital	123	38.8	286	34.2
Private medical centre/hospital	58	18.3	162	19.4
Other Hospital	6	1.9	3	0.4
Health Centre	3	0.9	12	1.4
Professional				
Specialist doctor	221	69.7	N/A	N/A
Resident doctor	48	15.1	N/A	N/A
Resident + Specialist	28	8.8	N/A	N/A
Nurse	13	4.1	N/A	N/A
Audiologist	5	1.6	N/A	N/A
Other	2	0.6	N/A	N/A
Total	317	100	836	100

Each compensated claim was categorised into one of nine primary complication types, as shown in Table 2. In 96 out of the 317 cases, a secondary event was also identified as an additional cause for compensation. Surgical/procedural complications accounted for 66.6% of primary and 25% of secondary events (Tables 2, 3).

**TABLE 2:** Event types leading to compensation in 317 Otorhinolaryngology patient injury cases during 2012–2023 in Finland.

Event types	Primary event		Secondary event^a^	
	*N*	%	*N*	%
Surgical/procedural complication	211	66.6	24	25.0
Inadequate or inappropriate treatment or diagnosis	77	24.3	52	54.2
Medication or diagnostic agent	12	3.8	1	1.0
Delay	6	1.9	1	1.0
Other	5	1.6	9	9.4
Diagnostic error	3	0.9	2	2.1
Infection	2	0.6	3	3.1
Anaesthesia	1	0.3	1	1.0
Device failure	0	0	3	3.1
Total	317	100	96	100

^a^ In 96 cases (30%), there were two significant reasons that lead to compensation.

**TABLE 3:** Categorisation of the surgical and procedural complications of ORL‐HNS related patient injuries into 11 distinct subcategories.

Procedural complications	Complication	
	*N*	%
Damage to adjacent structure	67	31.8
Nerve injury	46	21.8
Infection	26	12.3
Re‐operation	13	6.2
Inadequate surgical technique	9	4.3
RFA complication	9	4.3
Wrong‐site procedure	9	4.3
Other	9	4.3
Foreign body	8	3.8
Local anaesthesia	8	3.8
Unreasonable harm	7	3.3
Total	211	100

In the category of inadequate or inappropriate treatment or diagnosis (24.3% of primary events and 54.2% of secondary events), cases included instances where proper diagnostic tests were not conducted (e.g., necessary imaging was not performed), treatment was insufficient (e.g., a necessary surgery was not performed) or the treatment was inappropriate (e.g., unnecessary surgery was performed). Other unspecified events included system errors (*n* = 7), neglect of latex allergy (*n* = 1), the results not communicated to patient (*n* = 1), diagnostic delay (*n* = 1), surgical risks not discussed (*n* = 1), accidental refracturing of a rhinoplasty nose (*n* = 1), incorrect tracheostomy inner cannula ordered causing hazard (*n* = 1), and arm fracture sustained from a fall during transfer to the operating table (*n* = 1).

The 211 surgical and procedural complications were further divided into 11 more detailed subcategories (Table 3). The most common complications involve unintentional damage to nontargeted tissues, such as nerves or other adjacent structures, as well as infections. Additionally, 43 patients (20%) experienced a second significant procedural complication: 26 required reoperation (12.3%), 12 developed an infection (5.7%), four sustained a nerve injury (1.9%) and one suffered damage to an adjacent structure (0.5%). Unreasonable harm is defined by the PIC as a consequence that is significantly disproportionate to the illness or injury being treated, as well as to the patient's overall health or condition.

Table 4 displays 10 procedures that were most often linked to compensated patient injuries between 2013 and 2022 and their amounts compared with the total number of each procedure performed during that period in Finland. The 2013–2022 period, instead of the 2012–2023 period, was chosen because detailed data on the total number of procedures performed were available for these years only on the basis of statistics from the Finnish Institute for Health and Welfare.

**TABLE 4:** Top 10 compensated ORL‐HNS complications, procedures performed and frequency per 1000 procedures (2013–2022).

Procedure	Compensated complications	Total amount of procedures	Compensated complications per 1000 procedures
	*N*	*N*	
Endoscopic sinus surgery (FESS)	23	32 743	0.70
Tonsillectomy	21	49 013	0.43
Septoplasty	12	16 643	0.72
Single cervical lymph node removal	12	6785	1.77
Partial removal of the parotid gland	7	4992	1.40
Stapedotomy	5	2922	1.71
Adenoidectomy	5	40 459	0.12
Myringoplasty	4	4910	0.81
Adenotonsillectomy	4	13 930	0.29
Tympanostomy	3	88 593	0.03

The highest number of compensated surgical/procedural claims came from FESS surgery (23 compensated claims, with a compensation ratio of 0.7 per 1000 procedures) and septoplasty (12 compensated cases, with a compensation ratio of 0.72 per 1000 procedures). In contrast, the highest compensation rates were observed for procedures such as single cervical lymph node removal (1.77 per 1000 procedures) and partial removal of the parotid gland (1.4 per 1000 procedures). Among the three most common procedures—tympanostomy, tonsillectomy and adenoidectomy—the compensation ratios were 0.03, 0.43 and 0.12 per 1000 procedures, respectively. Seventeen compensated complications related to thyroid surgery were excluded from Table 4, as these procedures are predominantly performed by general or endocrine surgeons in Finland. Consequently, procedure‐specific data for ORL‐HNS were not available for analysis.

We also investigated whether gender had a significant effect on the likelihood of compensation and found no significant difference (*p* = 0.143). Additionally, we examined whether age was correlated with the likelihood of compensation and found a significant association (OR 0.992, 95% CI 0.985–0.999, *p* = 0.016), indicating that each additional year of age slightly decreased the odds of compensation. The likelihood of compensated claims did not differ significantly between university hospitals and regional hospitals (*p* = 0.156). Of the 499 complaints filed concerning university hospitals, 127 were compensated and 372 were not. In contrast, 408 complaints concerned regional hospitals, with 122 compensated and 286 not compensated.

The number of complications per subspecialty was as follows: rhinology, 97 (31%); head and neck surgery, 95 (30%); laryngology, 47 (15%); otology, 45 (14%); paediatric, 23 (7%); and audiology, 10 (3%). The average compensation was €10 031 and the median was €4056 (range €35–180 903). Compensation ranged from minor temporary harms to severe permanent injuries.

## 4 Discussion

This study provides an overview of ORL‐HNS patient injuries in Finland over a 12‐year period (2012–2023). The most common compensated injuries were surgical complications, including damage to adjacent structures and nerve lesions, and inadequate or inappropriate treatment or diagnosis. These findings align with the results of the previous research by Lehtivuori et al. [9] and Navaratnam et al. [4], both of whom similarly identified surgery‐related injuries as the predominant category of malpractice claims.

Compared with that in the previous decade, the distribution of compensated ORL‐HNS complications remained consistent. A total of 233 cases were compensated in Finland between 2001 and 2011, with 84.3% of these linked to surgical procedures [10]. Rhinology accounted for 36% [11], otology accounted for 19% [12] and paediatric accounted for 7% [13]. In England (2013–2018), head and neck surgery dominated claims at 43%, followed by otology and rhinology at 24% each [4].

Unlike in many other countries, Finland's compensation system emphasises reimbursement based on actual economic impacts, such as sick leave and functional impairment. The Finnish healthcare and patient injury compensation systems are quite like other Nordic countries. The compensation amounts are determined based on the provisions of the Tort Liability Act [14] and the guidelines issued by the Traffic and Patient Accident Board [15]. In this study, the average compensation was €10 031, with a median of €4056 (range €35–€180 903). Contrary to Lehtivuori et al. [9] article, where the average was $4097, with a median of $1904 (range $168 to $27022). In contrast, in the US, medical malpractice claims in ORL‐HNS average $1.8 million, with out‐of‐court settlements averaging $545 000 per case [16]. In the UK, litigation involving English health trusts from 2008 to 2018 incurred £87 million for 612 claims [17].

Despite advances, complication rates have remained stable, indicating persistent inherent risks. Previously, 33% of complications originated from university hospitals, 54% from regional hospitals and a small percentage from private practise [9]. In our study, the distribution was more balanced, with 40.1% of complications occurring in university hospitals and 38.8% occurring in regional hospitals. This shift is likely due to the centralization of high‐risk procedures, particularly head and neck surgeries, in university hospitals. During the study period, cancer surgeries in Finland were centralised from regional hospitals to university hospitals, further contributing to this trend.

Furthermore, our analysis demonstrated that increasing patient age is associated with a slight decrease in the likelihood of receiving compensation, whereas the type of hospital (university vs. regional) had no significant impact. These findings suggest that in ORL‐HNS, risk factors for patient injuries are more closely tied to procedural complexity than to patient demographics or healthcare settings. However, the centralisation of high‐complexity cases in university hospitals may confound these results, given the increased likelihood of complications inherent to such procedures. In support of these findings, studies have demonstrated that patients with head and neck squamous cell carcinoma treated at high‐volume facilities have better outcomes than those treated at lower volume centres [18]. The same findings were reported in studies regarding advanced laryngeal cancer [19, 20].

Notably, there is a concentration of surgical complications among specialists, which contrasts with other surgical fields, such as orthopaedics, where residents often perform more procedures, especially during on‐call shifts [9]. ORL‐HNS surgeries, however, are typically conducted during regular business hours by specialists, which may contribute to the distinct pattern of complications in this field. This underscores the importance of maintaining rigorous safety standards even in routine surgical procedures. Notably, in this study, there were many incorrect site procedures. Wrong‐site procedures remain ‘never events’, reinforcing surgical checklist use.

A noteworthy trend found in this study is the increasing incidence of reported patient injuries from procedures conducted in private practise, which were previously close to 7% and are now up to 18.3%. This could reflect a growing private sector, with more procedures being performed outside the public healthcare system. According to the THL data, the percentage of procedures performed by private providers out of all surgeries in Finland has shown an increasing trend, increasing from 1.33% in 2015 to 3.45% in 2024 [21]. This raises important questions about the consistency of care and safety standards across different healthcare settings, particularly as the private sector continues to expand.

One of the main strengths of this study is the use of data from the PIC. Operating under a no‐fault mode, the PIC allows patients to receive compensation without litigation and the need to prove negligence, streamlining the claims process and reducing the emotional and financial burden often associated with fault‐based systems. The PIC handles all claims of both public and private healthcare settings, ensuring fair treatment for all patients. Our findings also indicate that claims are processed equitably, as gender does not significantly affect the likelihood of compensation. Additionally, the PIC's centralised and uniform approach minimises administrative costs and speeds up their resolution time.

This study has several limitations. While the dataset is broad and inclusive, it does not account for unreported injuries, a common limitation in studies of this nature. Therefore, our data do not provide insights into the overall risk of specific procedures. Complications in more demanding and complex procedures are often anticipated and therefore less likely to result in compensation. Conversely, complications in routine procedures may be perceived as deviations from expected outcomes, making them more noticeable and more likely to lead to injury claims. This heightened expectation for flawless outcomes in routine surgeries could contribute to increased reporting and compensation rates. The retrospective nature of the study limits the ability to establish causal relationships.

## 5 Conclusion

The number of compensated patient injuries in ORL‐HNS has remained low and has shown little variation over time, but the compensation amounts have increased. The compensated cases predominantly involved specialists working in regional hospitals or university clinics. The most frequently compensated procedures were endoscopic sinus surgery (FESS), tonsillectomy, septoplasty and single cervical lymph node removal. The overall distribution of claims across subspecialties has remained relatively constant over time.

## Author Contributions

Alexander Westerholm and Morag Tolvi designed the study and collected and analysed the data. Alexander Westerholm drafted the manuscript. Satu Lamminmäki, Lasse Rämö, Leena‐Maija Aaltonen, Paula Virkkula and Antti Mäkitie revised the manuscript. All authors approved the final version and take responsibility for the content.

## Funding

The corresponding author has received funding from the Finnish medical society (Finska Läkaresällskapet).

## Ethics Statement

The study protocol was approved by the institutional review board of the PIC. As a retrospective registry‐based study with no patient intervention, approval from a medical ethics committee and individual informed consent were not required under Finnish legislation (Medical Research Act 488/1999).

## Conflicts of Interest

The authors declare no conflicts of interest.

## Data Availability

The data analysed during the current study are not publicly available due to patient confidentiality but are available from the corresponding author upon reasonable request.
